# Three-year delay in diagnosis of pulmonary sarcoidosis due to presence of necrotizing granulomas: a cautionary case report

**DOI:** 10.3389/fmed.2024.1464493

**Published:** 2024-11-19

**Authors:** Yubing Yue, Rao Du, Ding Han, Tianxia Zhao, Chunfang Zeng, Yinhe Feng

**Affiliations:** ^1^Department of Respiratory and Critical Care Medicine, Deyang People's Hospital, Affiliated Hospital of Chengdu College of Medicine, Deyang, China; ^2^Department of Respiratory and Critical Care Medicine, West China Hospital, Sichuan University, Chengdu, China

**Keywords:** pulmonary sarcoidosis, puncture, biopsy, granuloma, case report

## Abstract

Diagnosis of pulmonary sarcoidosis can be difficult and strongly dependent on clinical experience, especially when necrotizing granulomas are present. Here we report an individual who, 3 years after onset of symptoms, was definitively diagnosed with pulmonary sarcoidosis based on percutaneous lung biopsy under the guidance of computed tomography, after he failed to receive a specific diagnosis at other tertiary hospitals based on cervical lymph node biopsy and transbronchial needle aspiration under the guidance of endobronchial ultrasonography. After his definitive diagnosis at our medical center, he was given corticosteroids, which led to remission. Clinicians, especially in areas lacking suitably experienced pathologists, should be aware of how to diagnose sarcoidosis in the presence of abundant necrotizing granulomas in order to ensure timely diagnosis.

## Introduction

Sarcoidosis is a systemic disease of unknown origin involving non-caseous, non-necrotizing epithelioid granulomas (1). The disease can vary substantially in its manifestations, affected tissues and response to treatment (2). Although it is self-limiting in most patients, approximately one quarter of patients may suffer a progressive, chronic course leading to irreversible injury, such as pulmonary fibrosis, cirrhosis, fatal arrhythmia, or blindness (3). Incidence around the world varies from ~2 to 11 cases annually per 100,000 people, and 90% of cases involve the lungs, with smaller proportions involving the skin or eyes (4). In recent years, bone marrow involvement have also been rarely reported in pediatric and adult sarcoidosis (5). More than 10% of cases of pulmonary sarcoidosis involve the pulmonary parenchyma as well as intrapulmonary and peripheral lymph nodes (6).

Pulmonary sarcoidosis is diagnosed by exclusion and based fundamentally on histopathology, typically of tissue from a superficial, easy-to-biopsy site as well as tissue from the affected area in the chest (7). Histopathology should indicate non-caseous, non-necrotizing epithelioid cell granulomas. However, the granulomatous-type inflammation that is also found in other pathological conditions often makes the diagnosis difficult and may confuse pathologists into misclassifying sarcoidosis as another disease.

Here we describe the case of an individual in China who, 3 years after onset of symptoms, was definitively diagnosed with pulmonary sarcoidosis involving the peripheral lymph nodes, liver and spleen after his diagnosis went undetected at other tertiary hospitals. We attribute the missed diagnosis to the presence of abundant necrotizing granulomas, which distracted pathologists from the true underlying condition. Clinicians and pathologists should be aware of the potential of sarcoidosis in the presence of necrotizing granulomas and which techniques may be more reliable for diagnosing the condition.

## Case presentation

A 50-year-old Chinese man came to our tertiary hospital complaining of cough and progressive dyspnea lasting longer than 3 years, as well as dryness of the eyes and cervical lymph node enlargement lasting longer than 1 year. The patient reported no hemoptysis, chest pain, hot flashes, night sweats, or weight loss. During the previous 18 months, he had visited two other tertiary hospitals in western China. At the first hospital, histopathology of neck lymph node puncture indicated granulomatous inflammation with necrosis, and the patient was referred to the second hospital for further diagnostic work-up. At the second hospital, a chest computed tomography revealed regional lymphadenopathy, diffuse nodules in the lungs, and a mass in the right lower lobe. He then underwent transbronchial needle aspiration under the guidance of endobronchial ultrasonography (EBUS-TBNA), which indicated the same pathological results as the first hospital. Histology based on acid-fast, periodic acid Schiff, or hexamine silver stains were unremarkable, while a polymerase chain reaction test for the presence of *Mycobacterium tuberculosis* was negative. The patient was discharged from the hospital without diagnosis or treatment.

When he was admitted to our hospital, the patient reported no recent travel; no history of infectious or chronic diseases, trauma or clinical procedures other than the diagnostic procedures described above; and no history of alcohol or drug use. He reported having smoked at least 20 cigarettes per day for 30 years, then quitting more than 1 year before admission to our hospital. For 6 months prior to admission, he had been taking pregabalin (75 mg) twice daily to treat post-herpes neuralgia in his left chest.

At admission, the patient had normal body temperature (36.3°C), respiratory rate (19 breaths/min), heart rate (94 beats/min) and oxygen saturation in ambient air (97%), but his blood pressure was high (144/102 mmHg). Nothing remarkable was found on physical examination, routine blood tests, or assays of hypersensitive C-reaction protein, brain natriuretic peptide, liver function, coagulatory or connective tissue disease-associated antibodies, electrolytes or cardiac markers in serum, gases in arterial blood, or interferon-γ release. However, the patient showed elevated uric acid in serum (516.6 μmol/L).

**Figure 1:**
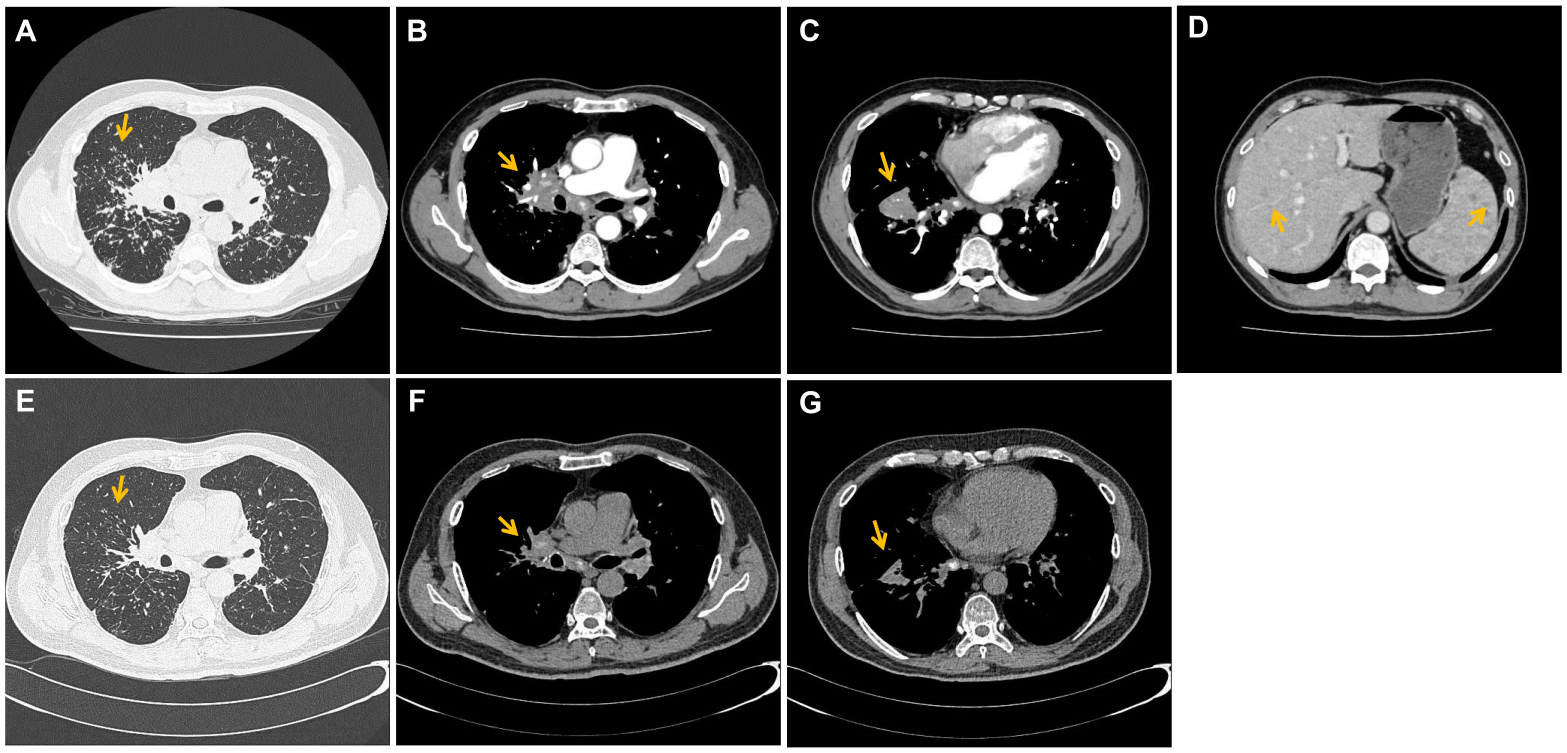
The initial and follow-up CT images of patient. **(A)** Computed tomography (CT) of the chest displayed diffuse nodules and consolidation in the lungs (yellow arrow). **(B)** Additionally, enlarged axillary and paraspinal lymph nodes with calcification and mild degree of enhancement were found in the bilateral mediastinum (yellow arrows); **(C)** as well as a mildly enhancement mass (about 5.5 × 3.4 cm) where a CT-guided percutaneous lung biopsy was performed (yellow arrows); **(D)** diffuse nodules in the liver and spleen were also detected (yellow arrows); **(E–G)** were after 2 months treatment. The condition of nodules, consolidation, lymphadenopathy and the mass showed significant improvement (yellow arrow).

Enhanced chest computed tomography displayed diffuse nodules and consolidation in the lungs. Additionally, enlarged axillary and paraspinal lymph nodes with calcification were found in the bilateral mediastinum. A mildly enhanced mass measuring 5.5 × 3.4 cm in the anterior basal segment of the right lower lobe and diffuse nodules in the liver and spleen were also detected (Figures 1A–D).

**Figure 2:**
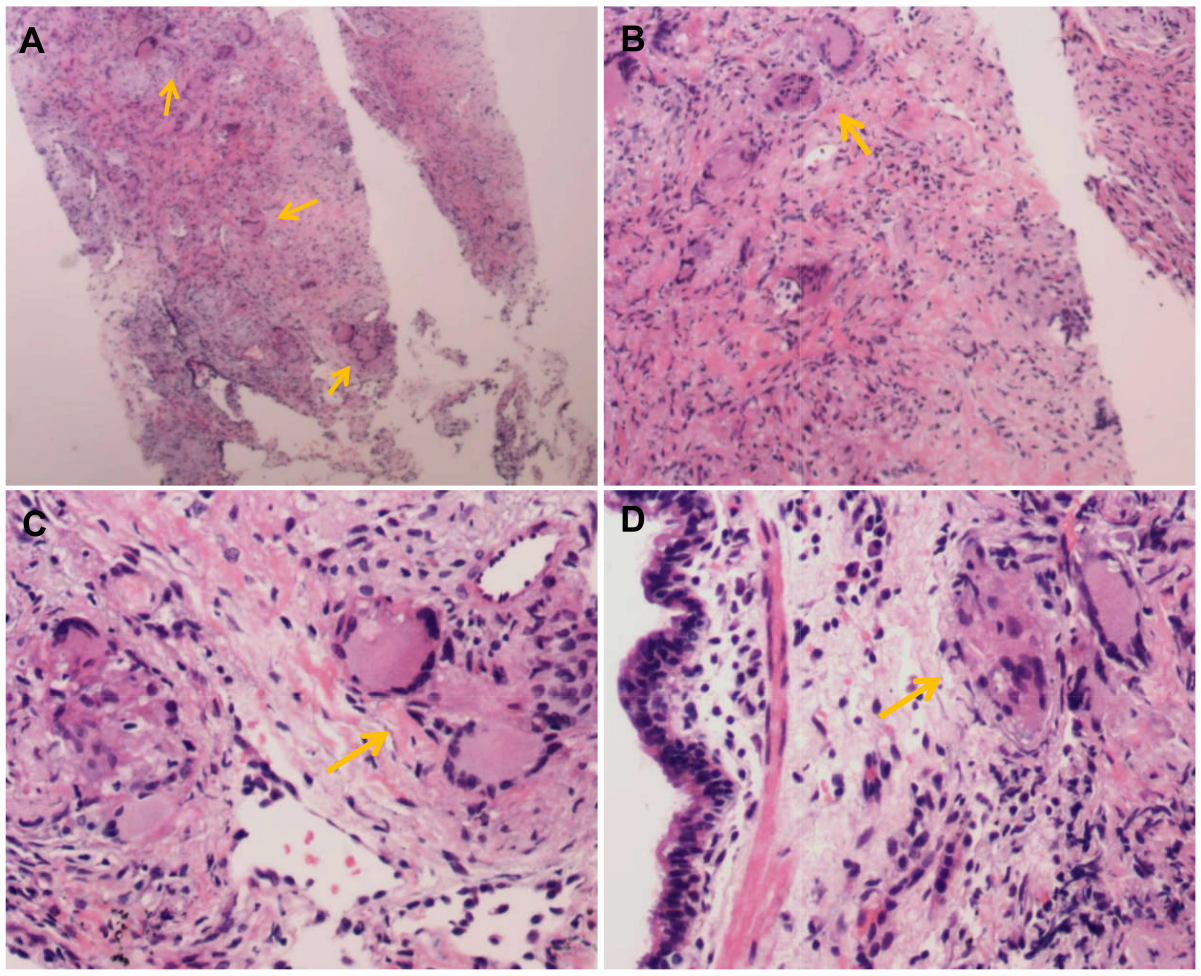
The histopathological staining of the lung sample. Tissue biopsy with multiple non-necrotizing granulomas (yellow arrow) at different magnification stained with hematoxylin-eosin (**A**: 40×), (**B**: 100×), (**C**: 200×), and (**D**: 200×).

To rule out lung cancer, the mass underwent percutaneous lung biopsy under the guidance of computed tomography, which indicated granulomatous inflammation (Figure 2). Histology of the biopsy after periodic acid schiff or hexamine silver staining was unremarkable. Based on the above findings as well as the results of ophthalmological tests and abdominal computed tomography, the patient was definitively diagnosed with pulmonary sarcoidosis involving the liver, spleen, and peripheral lymph nodes.

The patient was prescribed oral prednisone at 30 mg daily. After 1 month on this therapy, the patient reported substantial improvement in coughing and dyspnea. After 2 months of treatment, a follow-up chest computed tomography showed significant improvement in the condition of nodules, consolidation, lymphadenopathy and the mass (Figures 1E–G). The patient was switched to oral prednisone at 20 mg daily, and follow-up 1 month later showed no evidence of sarcoidosis recurrence. The patient was switched to prednisone maintenance therapy at 10 mg daily.

## Discussion

Clinical symptoms, computer imaging and physiological investigations of sarcoidosis are lack of specificity. Approximately one third of individuals with active sarcoidosis present non-specific manifestations such as fatigue, low fever, weight loss, night sweats, and joint pain (8). Hypercalcemia, even malignant hypercalcemia, is one of its manifestations (9). Dry cough, shortness of breath and chest pain are relatively common manifestations of pulmonary sarcoidosis (8), consistent with our patient's presentation of cough and progressive dyspnea, but respiratory symptoms may be neglected during diagnosis if the primary manifestations involve tissues other than the lungs. The causes and drivers of sarcoidosis remain unclear, and numerous factors have been implicated, including infection, dust, antigen-presenting cells, CD4^+^ T cells, cytokines such as interleukin-2 and tumor necrosis factor-*A*, as well aspolymorphisms in genes encoding human leukocyte antigen and butyrophilin-like protein 2 (3).

Sarcoidosis is a systemic granulomatous disease that may affect all organs but preferably lungs and lymph nodes. Some of the organs involved are hidden and not easily detected. Although a recent meta-analysis study suggested that the sensitivity and specificity of positron emission-computed tomography (PET-CT) in the diagnosis of pulmonary sarcoidosis were 0.971 and 0.873, respectively, PET-CT also does not ensure identification all organ involvement of pulmonary sarcoidosis (10). Our patient showed bilateral hilar lymphadenopathy and pulmonary infiltrates consistent with Scadding stage II in pulmonary sarcoidosis (11). Pulmonary sarcoidosis more often involves the upper lobe of both lungs and shows diffuse nodules, but our patient had a mass in the right lower lobe, which led us to examine the possibility of lung cancer. Our patient showed macronodules, which presumably arose through coalescence of granulomata and indicate more extensive disease (12), consistent with the involvement of multiple organs in our patient. Similarly, Marc et al. reported a rare pulmonary sarcoidosis case presenting with a large, solitary lung mass with imaging features of lung cancer (13). In addition to rare lump-like image findings as our patient, non-specific interstitial pneumonia (NSIP) lookalike pattern have recently been reported as a distinct pattern of pulmonary sarcoidosis on high-resolution computed tomography (HRCT) (14).

Our patient showed no signs of liver dysfunction but did show diffuse miliary-like nodules in the liver and spleen as well as enlargement of the spleen. The liver is involved in ~20% of cases of intrathoracic sarcoidosis (15), though autopsy-based studies suggest the real incidence may be as high as 80% (16). When the liver is affected, the spleen also tends to be affected. Hepatic sarcoidosis typically manifests as no symptoms or as mild, non-specific symptoms such as abdominal discomfort, exertion, vomiting, weight loss and fever, which is consistent with the presentation in our patient. Computed tomography of the liver and spleen typically reveals hepatosplenomegaly, diffuse nodules and rare solitary nodules, which are not specific for sarcoidosis. Up to 15% of patients with pulmonary sarcoidosis show spleen enlargement or splenic nodules on abdominal computed tomography (17). Diffuse splenic nodules associated with extensive tissue involvement outside the lungs and predicts worse prognosis (18), which may appear in our patient at longer follow-up.

Pulmonary sarcoidosis is a difficult condition to diagnose, and its diagnosis remains one of exclusion. Biopsy of peripheral lymph nodes and EBUS-TBNA failed to detect in our patient the non-necrotizing granulomas composed of epithelioid histiocytes and multinucleated giant cells, surrounded by palisading lymphocytes, plasma cells and fibroblasts, which are the hallmark of sarcoidosis (11). We attribute this failure to the abundance of necrotizing granulomas in the tissue sections, which distracted the pathologists and perhaps because of their lack of experience led them to focus more on the possibility of fungal or mycobacterial infection. Thus, our case highlights the need for clinicians and pathologists to consider the possibility of sarcoidosis even in the presence of abundant necrotizing granulomas. Indeed, one study has suggested that about 20% of cases of sarcoidosis involve some degree of necrotizing granulomas in biopsies (19). The riskier technique of percutaneous lung biopsy under the guidance of computed tomography did detect non-necrotizing granulomas in our patient and, after our careful exclusion of infection through appropriate histological stains, allowed us to diagnose him with pulmonary sarcoidosis. While sarcoidosis is the most frequent cause of granulomatous disease (whether necrotic or not), the second and third most frequent causes are tuberculosis and sarcoid reaction due to malignancy (20), which should therefore be considered during differential diagnosis. A rare finding, non-necrotizing granulomas formation in bone marrow biopsy have also been reported in Brucellosis (21). In some cases, other rare granulomatous diseases, such as drug-induced granulomatosis, Crohn's disease, granulomatosis with polyvasculitis, and eosinophilic granulomatosis need to be ruled out (22). Thus, the accuracy of the diagnosis involves a sensible differential for alternative diagnoses of infectious or non-infectious diseases.

The therapeutic management of pulmonary sarcoidosis is challenging due to the heterogenous of the clinical comorbid conditions, response to therapy and prognosis, thus should be individualized for each patient. Prednisone at an initial dose of 20–40 mg daily is the corticosteroid most frequently used to treat sarcoidosis. Daily doses above 40 mg appear not to provide additional benefit and instead increase risk of side effects (23). More recently, one study has used the proportions of circulating PD-1^+^ CD4^+^ memory T cells and PD-1^+^ regulatory T cells to predict treatment response to prednisone in pulmonary sarcoidosis (24). In the absence of consensus guidelines on tapering off corticosteroid therapy, the dose is typically reduced gradually after 2–4 weeks of initial treatment, and maintenance therapy at a daily dose of 5–10 mg is usually continued for 6–24 months. Second-line treatments include immunosuppressive and cytotoxic drugs such as methotrexate, leflunomide, and azathioprine, while biological drugs can serve as third-line treatments (25). Recent evidence supports the potential therapeutic benefits of anti-fibrosis drugs such as nintedanib due to their ability to reduce lung inflammation (26). Steroid treatment of sarcoidosis can be a double-edged sword: while the disease rarely relapses if it resolves on own, it relapses in 37–74% of individuals within 3–6 months after they stop steroid therapy (25). Some effective evidence has been observed that inhaled corticosteroid maintenance after induced systemic corticosteroid therapy can reduce relapse, but mainly budesonide (27). The possible explanation could be that inhaled budesonide creates the systemic anti-inflammatory activity, which is induced by rapidly absorbed into systemic circulation. This is also an option to the maintenance therapy for our patient. Generally, patients should be followed up for at least 3 years after they stop steroid therapy, since recurrence beyond that point seems to be rare (25).

Our case highlights the complexity of diagnosing pulmonary sarcoidosis, especially when multiple tissues are involved and when necrotic granulomas are abundant. Infection and cancer should be carefully excluded through appropriate histological staining and other tests. Although riskier than other biopsy approaches, percutaneous lung biopsy under the guidance of computed tomography may be necessary to definitively diagnose difficult cases. Future research should devote more attention to the diagnosis and treatment of extrapulmonary sarcoidosis.

## Data Availability

The original contributions presented in the study are included in the article/supplementary material, further inquiries can be directed to the corresponding author.
